# IgA nephropathy: a novel pathway in immunopathogenesis dependent on the timing of Epstein-Barr virus infection

**DOI:** 10.3389/fimmu.2026.1792410

**Published:** 2026-06-02

**Authors:** Zdenek Novak, Milan Raška, Jiri Mestecky

**Affiliations:** 1 Department of Surgery, University of Alabama at Birmingham, Birmingham, AL, United States; 2 Department of Immunology, Palacky University Olomouc, Faculty of Medicine and Dentistry and University Hospital, Olomouc, Czechia; 3 Department of Microbiology, University of Alabama at Birmingham, Birmingham, AL, United States; 4 Department of Medicine, University of Alabama at Birmingham, Birmingham, AL, United States; 5 Laboratory of Cellular and Molecular Immunology, Institute of Microbiology Czech Academy of Sciences, Prague, Czechia

**Keywords:** EBV infection timing, Epstein-Barr virus, IgA glycosylation, IgA maturation, IgA nephropathy, socioeconomic status

## Abstract

IgA nephropathy (IgAN) is an autoimmune disease in which polymeric IgA1, with joining (J) chain and altered glycosylation of the hinge region (HR), acts as an autoantigen recognized by naturally occurring IgG antibodies, resulting in generation of nephritogenic immune complexes (IC) that deposit in the kidney mesangium. By these criteria, IgA in IC and mesangial deposits displays properties identical to those of IgA produced by plasma cells infected by Epstein-Barr virus (EBV). Although ~95% of adults worldwide are EBV infected, there are important differences in the timing of infection in countries and areas with marked dissimilarities in socio-economic conditions. Epidemiological data convincingly documented that early EBV infection in children who live in developing countries, or in economically disadvantaged areas, display a significantly reduced frequency of IgAN compared to individuals from economically developed countries in which IgAN has been recognized as the leading cause of glomerulonephritis. Because physiological maturation of the IgA system is normally delayed until adolescence, in young children EBV infects B cells of Ig isotypes other than IgA, as these are relatively sparse. This early infection induces EBV-specific immune responses that prevent later infection of IgA-producing cells at older ages when these cells are more plentiful.

## 1 Introduction

IgA nephropathy (IgAN) is an immune-complex disease in which circulating immune complexes (CIC) of restricted molecular properties are deposited in the glomerular mesangium with ensuing inflammatory consequences ultimately leading to the loss of glomerular function (1–5). A long-term search for antigens of microbial or food origin failed to identify any such material involved in formation of these CIC (6). However, recent extensive studies have demonstrated that IgAN is an autoimmune disease in which IgA, exclusively of the IgA1 subclass with altered molecular properties, serves as an autoantigen that is recognized by ubiquitous, naturally occurring antibodies, predominantly of the IgG isotype, leading to the generation of nephritogenic immune complexes (IC) (7–10). Detailed analyses of these CIC and mesangial deposits revealed that IgA is exclusively of the IgA1 subclass, of polymeric (p) form with joining (J) chain, preferentially associated with lambda (λ) light chains, and deficient in galactose (Gal) in some of the short *O*-linked glycan chains in the unique hinge region (HR) of the heavy chains (7–21). The validity of this concept has been established by *in vitro* as well as *in vivo* studies (22, 23). Thus, CIC isolated from the sera of IgAN patients, or generated *in vitro* using Gal-deficient (Gd) pIgA1 and IgG from IgAN patients or healthy controls, displayed the nephritogenic activity (19, 23, 24). Furthermore, injection of human Gd-pIgA1 and polyclonal or monoclonal IgG antibody into immunodeficient mice induced mesangial deposition with proteinuria and hematuria (22), confirming the essential role for pGd-pIgA1 as an autoantigen in IgAN.

Although identification of Gd-pIgA1-J-λ as an autoantigen was for the first time revealed in our previous studies (7–9), the intracellular events involved in the production of IgA of these properties by plasma cells have remained enigmatic.

Surprisingly, the molecular properties of IgA in the CIC and mesangial deposits are absolutely identical to those of IgA produced *in vitro* by Epstein-Barr virus (EBV)-infected B cells that mature into IgA-secreting plasma cells (25–32). Thus, EBV-infected plasma cells selectively secrete IgA1 (28, 29) in polymeric form with J chain (30), preferentially with λ light chains (31), and with deficiency of Gal in the HR (32). This remarkable molecular identity of IgA in CIC and mesangial deposits with that produced by EBV-infected plasma cells prompted our comparative studies of EBV infection across populations stratified by age, ethnicity, sex, geographic location, timing of infection, and socioeconomic status to clarify its potential impact on IgAN pathogenesis. Although >95% of adults are globally infected with EBV, there are marked differences in the age at which infection occurs related to geographical area and socio-economic conditions (33–48). Recent data indicate that in countries with highly developed economies and hygienic conditions there is marked delay in the timing of EBV infection, which occurs predominantly in adolescence (4, 33). This timing is true for almost all European countries, North America, Japan, South Korea, and parts of China and Australia (**Table 1**). In sharp contrast, in low- and middle-income countries (LMIC) with less advanced economies and compromised hygienic conditions - such as most of Africa, India, Pakistan, and rural Australia - children are infected with EBV within the 1–4 years of life, as diagnosed by seropositivity, but without apparent clinical manifestations (4, 45, 46). At this age, children display a physiological deficiency of circulating IgA, with an absence or only low levels of plasma IgA and low numbers of IgA-producing cells in lymphoid tissues (49–57). Ethnic differences have been reported in the US and Australia (40, 41, 58, 59). Indigenous Aboriginals living in Sydney exhibited an incidence of IgAN comparable to that of the immigrant White population; in sharp contrast, in the rural areas of Australia and New Guinea, IgAN is either unknown or extremely rare (59). Consequently, in young children from these populations EBV infects B cells of other surface Ig isotypes and induces humoral IgG dominated and cellular immune responses that effectively prevent subsequent infection of later-developing IgA^+^ B cells. This timing contrasts to that of EBV infection in economically developed countries that occurs predominantly in adolescence. In the absence of immune responses induced by earlier infection, EBV has an increased opportunity to infect IgA cells which in adults are more numerous than cells producing IgG or IgM (57).

**Table 1:** List of countries with their respective IgAN frequency, mean of maximum EBV seropositivity between ages 0.5–9 years, and gross national income per capita level (2010).

Country	Mean IgAN relative frequency score (1 - High, 5 - Low to No IgAN presence) Ref (60, 62, 69–72)	Mean EBV seropositivity of maximum between ages 0.5 and 9 years (%)	Gross National Income per Capita (2010)	EBV References
Sweden	1	40.8	High	(138, 174–176, 190)
France	1	45.7	High	(132, 138, 158, 159)
Netherlands	1	49	High	(169, 170)
Czech Republic	1	56.2	High	(154)
Denmark	1	60	High	(155)
Estonia	1		High	
Finland	1		High	
Germany	1		High	
Hungary	1		High	
Poland	1		High	
Portugal	1		High	
Spain	1		High	
Lithuania	1		Upper middle	
South Korea	1.5	92.2	High	(166)
Japan	1.6	80.6	High	(33, 125)
Australia	1.7	69.6	High	(58, 77)
China	1.8	76.7	Upper middle	(144–151)
Singapore	1.8	67	High	(158, 172)
Greece	2	53	High	(162)
Croatia	2	59.6	High	(152)
Belgium	2	72	High	(139)
Italy	2	81.9	High	(191)
Colombia	2		Upper middle	
Romania	2		Upper middle	
UK	2.2	57	High	(138, 147, 185, 186)
USA	2.3	61.5	High	(41, 92, 138, 141, 187–189, 192)
Taiwan	2.7	92.1	High	(177, 178)
Thailand	2.7	93.3	Upper middle	(165, 179, 180, 193)
Malaysia	2.8	100	Upper middle	(167)
Brazil	2.9	82.3	Upper middle	(138, 140, 141)
Austria	3		High	
Mexico	3	88.5	Upper middle	(138, 168)
Lebanon	3		Upper middle	
Montenegro	3		Upper middle	
Russia	3		Upper middle	
Serbia	3		Upper middle	
Uruguay	3		Upper middle	
Kuwait	3.3		High	
Iran	3.4	73.7	Upper middle	(164)
India	3.9	90	Lower middle	(163)
Canada	4	75	High	(142)
Bahrain	4		High	
Oman	4		High	
Jordan	4		Upper middle	
Macedonia	4		Upper middle	
Peru	4		Upper middle	
Morocco	4		Lower middle	
Saudi Arabia	4.1		High	
Pakistan	4.3		Lower middle	
Indonesia	4.5	92	Lower middle	(138, 194)
Sudan	4.5		Lower middle	
New Zealand	5		High	
United Arab Emirates	5		High	
Turkey	5	96.3	Upper middle	(181)
South Africa	5		Upper middle	
Bangladesh	5	51.4	Low	(137)
Nepal	5		Low	
Slovakia		47.4	High	(173)
Faroe Islands		93	High	(157)
Barbados		95	High	(138)
Chile		28	Upper middle	(143)
Cuba		73	Upper middle	(153)
Argentina		89	Upper middle	(136)
Zambia		59	Lower middle	(126)
Ghana		93.7	Lower middle	(45, 93)
Papua New Guinea		100	Lower middle	(171)
Solomon Islands		100	Lower middle	(171)
Vanuatu		100	Lower middle	(171)
Gambia		55.5	Low	(160, 161)
Ethiopia		82	Low	(156)
Uganda		94.1	Low	(138, 158, 182, 183)
Kenya		100	Low	(126)

In this report, we extended previous studies concerning the global frequency of IgAN (60–62) to additional countries, correlated the frequency of IgAN with the known timing of EBV infection in individuals of different ages (38, 43), and considered the previously overlooked impact of marked differences in socio-economic status.

These studies offer alternative and contemporary pathways involved in immunopathogenesis of IgAN in addition to the extensive genetic reports concerning the geographic and racial aspects of this disease (63–68).

## 2 Methods

### 2.1 IgAN frequency

This report includes comparative studies of EBV infection rates as documented by seroprevalence in different geographics areas in relationship to age, socio-economic, and racial contexts. IgAN data and frequency grouping are based on the natural history of IgAN reviewed by Schena and Nistor (60), supplemented by additional articles (62, 69–72). The reference for each country lists the most common primary glomerular disease (PGD) by order of frequency shown as the percentage of patients with a biopsy-proven diagnosis of PGD. Based on these data, IgAN frequency was divided to 5 levels: level 1, IgAN was the most frequent PGD (31% - 50%). Levels 2–4 were 21% - 30%, 10% - 20% and <10% of PGN accordingly. If a report indicated absence or did not include IgAN in a particular country (among other glomerulonephritidies), we assigned it to group 5 (absent). If multiple articles were available for a country, an average value was calculated regardless of location and time of data collection. A threshold of an average value of 2.5 (≤2.5 versus >2.5 for IgAN groups and no-IgAN group, respectively) was used to dichotomize the groups for comparative purposes.

### 2.2 EBV seroprevalence

EBV-related publications were identified through searches of the PubMed and Scholar Google websites as well as utilizing UAB’s library resources and exchange partnerships. In addition to database searches, manual review of reference sections in each paper was completed and potentially relevant articles selected. Age-dependent seroprevalence data related to a specific disease such as, but not limited to, nasopharyngeal carcinoma, Burkitt’s lymphoma, and HIV were excluded from this review.

We were able to collect EBV-seroprevalence data for 42 countries between 1957 and 2022 (summarized in **Table 1**). These data were based on immunoassays (including, but not limited to, ELISA, EIA, immunofluorescence, fluoro-bead multiplex assay, and Paul-Bunnell test), anti-EBV viral capsid antigen (VCA EBV) IgG or its historical equivalents such as human-derived cell line established from biopsy fragments and cell clumps of Burkitt lymphoma (EB-1 cells, EB-3 cells), or lymphocytes infected with QIMR-WIL EBV strain (QIMR-WIL cell). EBV seroprevalence based on IgG levels was abstracted from each selected articles text or tables or estimated from graphs when necessary. The following information was extracted from each study: country for which data were available, years of study, type of laboratory assay used to assess seroprevalence, and EBV seroprevalence for each age group described. Seroprevalence data were obtained for all countries presented in an article, even if those were referred from third-party sources. For comparative purposes, the maximal value of seroprevalence between ages of 6 months and 9 years was calculated in order to use “the best case” scenario and also to reduce potential data missingness. If multiple articles or populations in an article existed for the same country, the average values were calculated regardless of location and time of sample collection.

### 2.3 Gross national income per capita

Income data for the year 2010 for each country were downloaded from a spreadsheet from the World Bank website (63). Gross National Income per Capita (GNIpC) was calculated using the World Bank Atlas method and assigned to 4 categories (dollar values in year 2010): low-income economies, GNIpC $1,005 or less; lower middle-income economies GNIpC, $1,006 - $3,975; upper middle-income economies, GNIpC $3,976 - $12,275; and high-income economies, GNIpC, $12,276 or greater (63). For comparative purpose we have coded high income as “4” and low income as “1”.

### 2.4 Statistics

Figures and table were prepared using the R statistical package (R Core Team [2021], Vienna, Austria). The Mann-Whitney U-test was used to compare medians between the IgAN groups. Spearman’s correlation was used to correlate EBV seropositivity and GNIpC with IgA groups. Univariable logistic regression was used to calculate Odds Ratios (OR). P-value ≤ 0.05 was considered to be statistically significant.

## 3 Results

Although IgAN has been considered an autoimmune disease whose incidence is associated with marked geographic and genetic differences, additional possibilities revealed that other, previously not considered aspects may be involved (4, 60). Because the properties of IgA in CIC and mesangial deposits are in every respect identical to those of IgA produced by EBV-infected cells (4, 5, 7, 10, 25–48), we analyzed available data concerning the global frequency of IgAN and its relationship to EBV infection with associated socio-economic disparities and the age-dependent maturation of the IgA system. Based on these data, we propose that exposure to EBV at an early age, when the IgA immune system is physiologically immature, induces protective humoral responses that reduce the likelihood of a future EBV infection of IgA^+^ B cells (73), thereby markedly diminishing the risk of developing IgAN.

### 3.1 Geographic distribution of IgAN is related to the timing of infection with EBV relative to maturation of the IgA system

Results of Schena and Nistor (60) and extended in this report, document the marked differences in the frequency of IgAN in various countries. From these data, it is clear that the frequency of IgAN displays strong geographic differences, with higher frequency in countries where EBV infection is delayed until adolescence or later. Most importantly, socio-economic status is of greater importance than racial differences. In economically developed countries, the EBV-seropositivity is delayed relative to populations in countries or areas that are less economically advanced. For example, EBV-seropositivity in African Americans or Australian Aboriginals living in rural areas is higher than in those living in urban areas (58), and it occurs at a much earlier age. Furthermore, when EBV infection occurs in early childhood, the circulatory immune system displays physiological IgA deficiency with the reduced numbers of IgA^+^ B cells that are potentially infectable with EBV. This status of the IgA immune system differs from that in adolescents who have a mature IgA system with more IgA^+^ B cells amenable to EBV infection (49–57).

**Figure 1:**
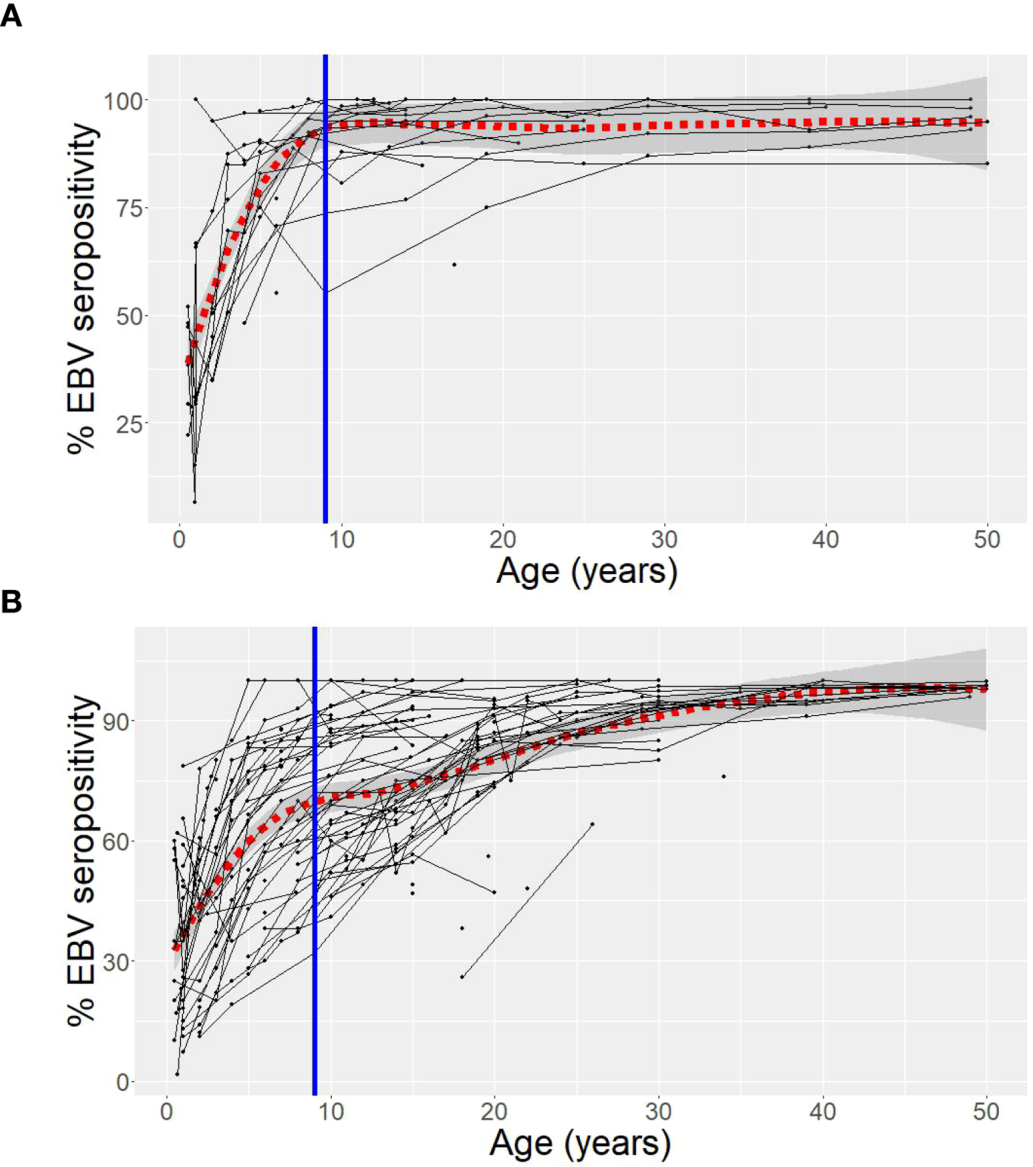
EBV seropositivity by age of persons in the countries with a low frequency of IgAN or no reported IgAN (average IgAN level >2.5) **(A)** and in the countries with a high frequency of IgAN (average IgAN level ≤2.5 with) **(B)**. Red line – Locally Estimated Scatterplot Smoothing method seropositivity *trendline*, Blue line – cut-off for 9 years of age (end of reference interval, 0.5–9 years). **(A)** EBV seropositivity by age in populations of countries with no reported IgAN or low frequency of IgAN. **(B)** EBV seropositivity by age in population of countries with high incidence of IgAN.

EBV-seropositivity was higher at an earlier age in countries with a low frequency of IgAN (moderate negative correlation r=-0.6, p=0.001, n=27) (**Figure 1**). For example, at the age of 9 years, the median (IQR) frequency of EBV seropositivity was 90% (75.0%-93.3%) in countries with a low frequency of IgAN compared to 60.7% (53.8%-75.5%) in countries with a high frequency of IgAN (p=0.001).

### 3.2 Frequency of IgAN and its relationship to the socio-economic conditions

**Figure 2:**
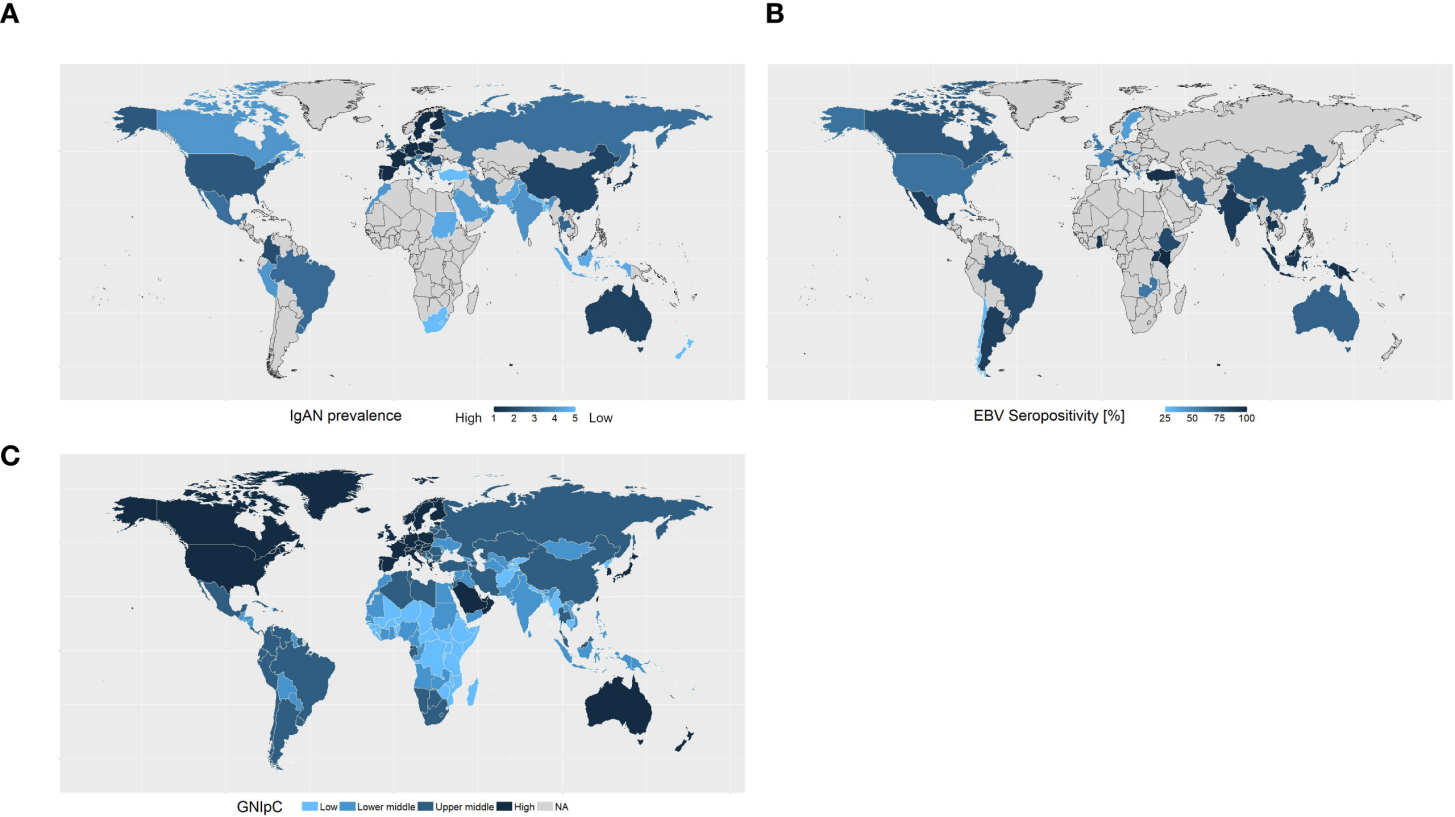
**(A)** IgAN frequency in the world (1-highest, 5-lowest). B) Mean of maximal EBV seropositivity for children between 6 months and 9 years of age. C) The world map of gross national income per capita (GNIpC); EBV map refs (33, 41, 45, 77, 92, 93, 126, 132, 135–189): IgAN map refs (61, 69–71): Gross national income per capita map ref (63) **(A)** IgAN frequency **(B)** Maximal EBV seroprevalence for children ages 0.5–9 years **(C)** The world map of gross national income per capita (2010).

The timing of EBV infection and frequency of IgAN may be related also to factors affecting hygienic conditions, including family income, number of siblings, shared living quarters and utensils, regional living customs, and frequency of breastfeeding (74). Comparative data on different ages of EBV infection, related to variability in socio-economic conditions in various countries and racial groups, further support our hypothesis (**Figure 2**). The higher frequency of IgAN occurs mainly in countries with high socio-economic standards and a delayed age of EBV primoinfection that is associated with a mature state of the IgA system attained in adolescence. Association of low socio-economic status with early EBV infection has been well established in the literature (38, 45, 46, 58, 75). Regarding the relationship of IgAN with socio-economic status, countries with a high frequency of IgAN fall mostly into high-income groups (medium positive correlation r=0.6, p<0.001 (all countries where IgAN and GNIpC is available, n=57) or high positive correlation r=0.8, p<0.001 (countries where also EBV data is available, n=27)) with income median (IQR) of 4 (4–4), in contrast to countries where the frequency is low or absent with median (IQR) of 3 (3, 4), p<0.001 (**Table 1**). .

Odds ratios for GNIpC association to IgAN (n=57) and EBV seropositivity association to IgAN (n=27) calculated using univariate logistic regression were 10.1 (p<0.001) and 0.9 (p=0.008), respectively. The adjusted ORs were 17.5 (p=0.025) and 0.9 (p=0.074) respectively. However, there is low negative correlation between EBV seropositivity and GNIpC (r=-0.4, p=0.008, n=42).

There are, however, observations that have not been considered previously. Almost all native Australian and New Guinea Aboriginals living in rural areas are infected by EBV within the first 2 years of life, due to unfavorable socio-economic conditions (58). IgAN has not been diagnosed in these situations, although biopsy data and information on the occurrence of other glomerulonephritis are available (76). In contrast, Aboriginals living in Sydney display a frequency comparable to the immigrant White population in which delayed EBV infection and a higher frequency of IgAN coincide (58, 77, 78). Prior to the age of 6 months, most infants are seropositive due to prenatal transplacental transfer of IgG antibodies from mothers, who by the time of pregnancy are >90% EBV-infected and seropositive (46). After 6 months, EBV-seropositivity is due to the EBV infection (46).Thus, differences in age at the time of EBV infection are related to variability in socio-economic conditions in different locations rather than race.

## 4 Discussion

Results in this report offer a novel view concerning the frequency of IgAN at the global level as related to the timing and impact of EBV primoinfection and to maturation of the IgA system with production of Gd-pIgA1-J-λ chains, and the importance of socio-economic factors in different regions (1, 2). Most importantly, the physiologically normal late maturation of the human IgA system (49–57, 79), relative to the time when EBV primoinfection occurs (40, 46), provides a rational explanation for the differences in frequency of IgAN (73).

Early EBV-infection, preceding the emergence of IgA B cells and plasma cells, leads to the early induction of humoral and cellular responses that prevent subsequent infection of other B cells, including IgA^+^ B cells or immature B cells that later switch to surface-positive (s) IgA^+^ B cells (47, 80, 81). Indeed, we have demonstrated that in EBV-seropositive White adults, a small proportion of sIgA^+^ B cells is infected with EBV, whereas in adult African Americans EBV-infected B cells do not display sIgA^+^ but instead are predominantly sIgD/M^+^ and sIgG^+^ B cells (25), supporting the importance of the timing of EBV infection.

**Figure 3:**
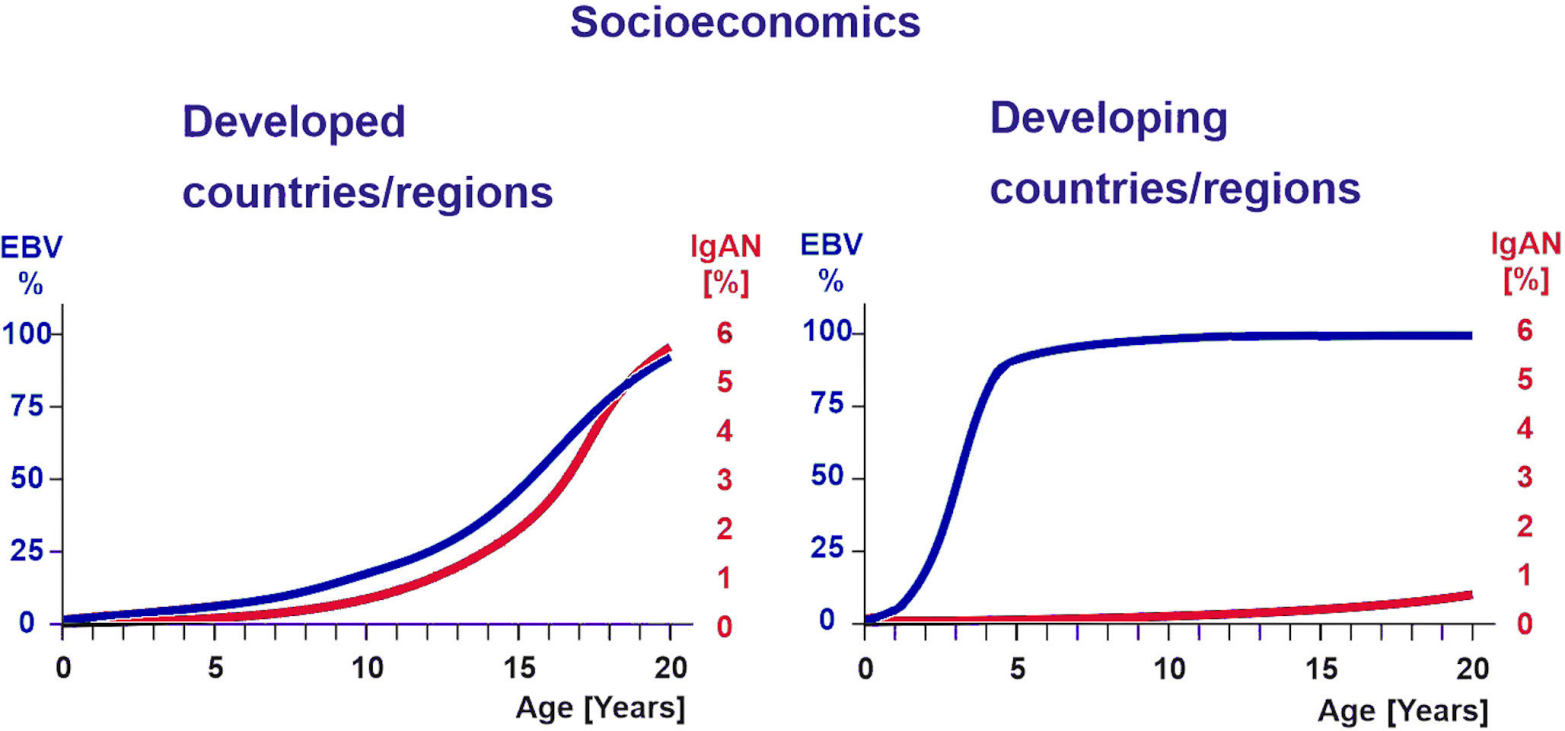
Proposed model of differences of IgAN prevalence (% is equal to cases per 10 000) and EBV seropositivity that vary by age and depend on the country’s socioeconomic status.

Despite earlier reports, IgAN is not the most common cause of glomerulonephritis globally (60, 61). The most important factor explaining the paucity of IgAN in African Americans and black Africans, some Asian, South American, and indigenous Australian populations is not their race (64, 65). Instead, widely differing socio-economic factors-when comparing countries with high income (HIC) and markedly better hygienic conditions, education, and per capita income, to those countries with low to middle income (LMIC), play the dominant role in the early low incidence of EBV infection (40, 41, 46, 73, 82) (**Figure 3**). Furthermore, in LMIC, prolonged breastfeeding with EBV-contaminated milk (74) is also likely involved in early infection and, thus, with a low incidence of IgAN (73, 83, 84). We propose that the immune responses ensuing from early EBV infection, at a time of physiological IgA deficiency, are of essential importance.

It may be argued that EBV-seropositivity, reaching ~95% of all adults irrespective of geography, would result in a higher frequency of IgAN. However, this position does not consider several relevant but frequently overlooked facts. Sera from healthy adults and from the relatives of IgAN patients who lack clinical manifestations also contain increased levels of Gd-pIgA1 and corresponding IgG antibodies (2, 9, 17, 85). IgAN patients display significantly higher levels of IgA, including Gd- IgA1, probably due to the production of EBV encoded viral IL-10 involved in the terminal differentiation of B cells into IgA1-producing plasma cells (86). Importantly the timing of EBV primoinfection may potentially be supported by the Ig isotype distribution of EBV-infected peripheral blood B cells: early infection, occurring before full maturation of the IgA compartment, would be expected to yield EBV-positive cells predominantly among IgD/M+ and IgG+ B cells, whereas delayed infection may permit detectable EBV infection of IgA+ B cells (25). Furthermore, the examination of kidneys from individuals who died violently or examined at the time of autopsy revealed IgA deposits in 4-16% even though these persons displayed no clinical evidence of kidney disease (87–90). The authors speculate that this may be due to the absence or low presence of anti-Gd-IgA1 IgG and C3 in mesangial deposits. Furthermore, the quantity, composition, and especially molecular mass of CIC play important roles in the clinical manifestation of IgAN (2, 20). The relative proportions of Gd-pIgA1 as the autoantigen and the corresponding IgG antibodies critically influence the biological activity of the CIC, a situation which bears analogy with serum sickness (91). As demonstrated earlier, the differences in molecular mass and size of CIC from IgAN patients or generated *in vitro* (19) determine the biological activity and play essential roles in immunopathogenesis of IgAN (2, 3).

The involvement of EBV in IgAN is further supported by several relevant findings. EBV, like other herpes viruses, establishes life-long, latent residence that evades elimination (34, 44, 47). Clinical manifestations are related to the magnitude of the induced immune responses and the age at which an individual is infected (40, 46, 92, 93). Surprisingly, the early EBV infection of children usually has no clinical manifestations (34, 37, 38, 48). However, upon effective stimulation, the virus may become re-activated with ensuing clinical consequences.

The most common EBV-dependent disease is infectious mononucleosis. Interestingly, and by analogy with IgAN, this disease is mostly unknown in Africa and other countries with low GNIpC, and it is 30-times *less* frequent in the African American population than in Caucasians, most likely due to the early induced humoral and T cell-mediated responses in children who tolerate this infection without obvious clinic manifestations (38, 43).

Among a broad list of drugs used in the treatment of patients with IgAN (94–96), dipyridamole has been given usually in combination with other agents to slow coagulation, dilate blood vessels, and reduce inflammation (97–102). Importantly, recent virological studies demonstrate that dipyridamole is also a potent inhibitor of EBV reactivation in infected human B cells (103). It is therefore conceivable that treatment of IgAN with dipyridamole prevents the reactivation of EBV in infected B cells, including those which, after terminal differentiation into plasma cells, secrete Gd-pIgA1 as the autoantigen in nephritogenic CIC.

Concomitant production of EBV and Gd-IgA1 in the same plasma cells may also explain Gal-deficiency in the HR of IgA1 due to the presence of numerous *O*-linked glycan chains on the main EBV glycoprotein, gp350 (104, 105). Limited cellular pools of UDP-Gal and β1,3-Gal transferase may lead to preferential galactosylation of viral gp350 instead of IgA1 (106). Furthermore, the EBV genome comprises a gene encoding viral (v) IL-10, which is structurally and functionally analogous to its human counterpart (107). IL-10 is not involved in the Ig isotype switching to sIgA^+^ B cells but is essential in the terminal differentiation of sIgA^+^ B cells into IgA-secreting plasma cells (86, 108); increased serum levels of IL-10 have been observed in IgAN patients (109, 110). Furthermore, EBV-encoded LMP1 cooperates with BAFF and APRIL to induce T cell-independent immunoglobulin class switching toward IgG and IgA (111). In turn, this process may lead to selectively elevated plasma levels of IgA1 in IgAN patients (112).

In the absence of suitable experimental animal models, data are inevitably obtained from individuals with IgAN and relevant controls including the healthy individuals and those with other types of glomerulonephritidies (7, 9). EBV exclusively infects humans (48). Except for hominoid primates, all commonly used experimental animals possess IgA that is analogous to human IgA2 rather than IgA1, and thus lacks a HR with *O*-linked glycans which are critical in the immunopathogenesis of IgAN (4, 10, 24).

EBV is associated with a broad spectrum of unrelated human diseases of infectious (e.g., infectious mononucleosis), autoimmune (e.g., multiple sclerosis, systemic lupus erythematosus, and inflammatory bowel disease) (113–120), or malignant (e.g., nasopharyngeal, gastric carcinomas, non-Hodgkin lymphoma and multiple myeloma) nature (121–130). Furthermore, there is considerable variability in the clinical nature of EBV-associated diseases, ranging from asymptomatic to severe clinical manifestations, as observed in infectious mononucleosis. IgAN is no exception. IgAN also displays highly variable clinical manifestations, from modest urinary abnormalities to the fulminant injury leading to kidney failure; in some persons, IgA deposits have been discovered at necropsy (88, 90) or kidney donation (89) in the absence of any clinical evidence of kidney disease (1, 131).

## 5 Limitations

The present data are associated with some inevitable limitations. Tests used for the determination of EBV seropositivity have varied during the dates included in this study. The Paul-Bunnell test used for a long time for diagnostic purposes of infectious mononucleosis has been replaced by several alternative but comparatively or even more reliable assays due to the simplicity and the cost-reduced ELISA currently used. Retrospective evaluations of IgAN patients have not included inquiries concerning EBV serology, socio-economic data including family income, education, or living conditions such as the number of individuals per room, sharing of utensils and hygienic conditions, all of which play an essential role in EBV infection (38–40). Furthermore, there are no data available as to socio-economic differences among countries with low GNIpC with respect to the frequency of IgAN. Also, the data concerning the frequency of IgAN in different countries, EBV seropositivity, and the Gross National Income are inevitably not uniformly derived from the identical time due to the wide range of publication dates. Thus the data were abstracted from alongside generally low availability of information for targeted age groups. In countries with a highly variable individual income such as India, there is no information concerning the incidence of IgAN in families in the high-income group as compared to the overwhelming majority of inhabitants on the low income spectrum living in inferior socio-economic and hygienic conditions. Available epidemiological data have convincingly demonstrated that the EBV infection in most of the countries with high level of socio-economic status is remarkably delayed. We propose that this finding may explain the higher incidence of EBV-associated diseases, including IgAN, in individuals with the developed IgA system at time of EBV primoinfection. Importantly, several authors predict the trend in the time-related delay in the EBV infection with the higher incidence of autoimmune and malignant diseases (33, 132). This concept provides further support for our hypothesis. Unfortunately, we have no relevant information from China because no data are currently available with respect to the differences in the socio-economic status of individuals living in rural or urban areas of China.

Also, for some countries, such as Japan, the available EBV data represent historical and geographically limited data that might not be representative of the whole country and might be misrepresenting overall status. However, a trend is observed where the level of EBV infection among 5–7-year-old children is decreasing over time as reported for years 1975-1995, likely correlating with increasing socio-economic level (33, 132).

Reasons for the higher incidence of IgAN in males vs. females remain unclear. Interestingly, epidemiological data indicate that girls become infected with EBV ~2 years earlier than boys, resulting in earlier protective immune responses. The unavailability of data concerning the incidence of IgAN and EBV seropositivity in children with differences in socio-economic status and living conditions (urban vs rural) and healthcare presents an additional limitation. For several countries (e.g., Russia), data concerning the relatively high incidence of IgAN (72) and economy are available, but the data concerning EBV seropositivity are not accessible.

## 6 Summary

In summary, we propose a novel pathway in the immunopathogenesis of IgAN. Based on the hitherto unconsidered role of EBV primoinfection in the production of Gd-pIgA1.J.λ that is identical to the IgA in CIC and mesangial deposits of patients with IgAN, the early age at EBV infection in populations in LMIC where IgAN is rare, and physiologically late maturation of the IgA system, we propose that IgAN is an autoimmune disease whose incidence is also strongly impacted by socio-economic conditions. Thus, IgAN can be added to the list of diseases of autoimmune character which are associated with EBV infection (39, 42, 113–120, 133, 134).

## Data Availability

The original contributions presented in the study are included in the article/supplementary material. Further inquiries can be directed to the corresponding author.
